# Congenital nasolacrimal duct fistula in Brown Swiss cattle

**DOI:** 10.1186/1746-6148-10-44

**Published:** 2014-02-18

**Authors:** Ueli Braun, Simon Jacober, Cord Drögemüller

**Affiliations:** 1 Department of Farm Animals, Vetsuisse-Faculty, University of Zurich, Winterthurerstrasse 260, CH-8057 Zurich, Switzerland; 2 Institute of Genetics, Vetsuisse Faculty, University of Bern, Bern, Switzerland

**Keywords:** Cattle, Nasolacrimal duct, Nasolacrimal duct fistula, Congenital anomaly

## Abstract

**Background:**

An increased incidence of nasolacrimal duct fistula in the offspring of dam J and three of her sons (bulls A, B and C) prompted a study to investigate the prevalence and clinical manifestation of this anomaly. The dam J, bull B, 255 direct offspring of bulls A, B, and C and eight other direct and indirect offspring of cow J were examined. The periocular region of each animal was examined for unilateral or bilateral nasolacrimal duct fistula and the location, appearance and size of the lesions.

**Results:**

Of 265 cattle examined, 54 had unilateral (n = 24) or bilateral fistula (n = 30). The prevalence of affected offspring differed significantly among the three bulls. The fistulae were located medial to the medial canthus of the eye and were 1 to 10 mm (median, 1 mm) in height and 1 to 12 mm (median, 2 mm) in length. The shape of the opening was circular in 58, oval in 23 and slit-like in three. One other animal had a large opening with an atypical shape and another had an abnormal medial canthus with several fistulous openings. Seventy openings were pigmented and 52 were hairless. The fistulae were clinically significant in 12 animals.

**Conclusions:**

The findings suggest a hereditary cause of nasolacrimal duct fistula in Brown Swiss cattle.

## Background

Congenital defects of the lacrimal apparatus are uncommon in humans and animals [1]. An understanding of the anatomy and embryological development of the lacrimal apparatus is paramount to the understanding of abnormalities [2,3]. Congenital defects may affect the lacrimal punctae and the lacrimal canaliculi proximally or the lacrimal sac and nasolacrimal duct distally [1]. Congenital anomalies of the nasolacrimal duct have been reported in horses and cattle [4-6], and abnormal openings of the lacrimal apparatus medial to the medial canthus of the eye have been described in humans and cattle [3,7]. The nasolacrimal anomalies reported in 13 Brown Swiss cattle in the USA [7] were abnormal openings located distal to the medial canthus of the eye. Some were easily seen and surrounded by hairless skin and others were not visible. Bilateral nasolacrimal fistula was reported in a Holstein calf [8] and in a five-year-old Brown Swiss bull [9]. The latter case was an artificial insemination bull (bull A) that had been referred to our clinic because of conjunctivitis. Examination of the bull revealed mucopurulent secretion from bilateral lacrimal fistula situated medial to the medial canthus of both eyes. Fluid infused through both lacrimal punctae escaped via the fistulous openings. Retrograde flushing of the nasolacrimal ducts resulted in the flush fluid flowing back out the nasal openings rather than the fistulous openings. The same findings were reported one year later in four other Brown Swiss cattle. Three of these were daughters of bull A, and the other was a daughter of his maternal half-brother (bull B), also used as an artificial insemination bull. The lesions were thought to be congenital. The primary goal of the present study was to determine whether this anomaly occurred in other offspring of bulls A and B and another maternal half-brother, bull C, which was also used as an insemination bull. Other goals were to describe the phenotypical appearance of any anomalies and to examine dam J and her direct and indirect offspring. Bull B, the only surviving bull of the three, was also examined.

## Methods

### Animals

A total of 265 cattle (263 females and 2 males) were examined for lacrimal fistula(e). This included the 19-year-old dam (dam J) of bulls A, B and C, bull B, 255 direct female offspring of bulls A, B and C and eight other direct (n = 5) and indirect (n = 3) female offspring of dam J (Table 1). The findings of bull A with bilateral lacrimal fistula were described previously [9]. Bull C had been slaughtered before the study and therefore could not be examined. The signalements of dam J and bulls A, B and C have been described [10].

**Table 1:** Distribution of 84 nasolacrimal duct fistulae in 54 offspring of dam J

**Animals**	**No. of affected animals**	**Nasolacrimal duct fistulae (n = 87)**		
		**Left**	**Right**	**Bilateral**
Pedigree cow J (n = 1)	1	1	–	–
Bull B (n = 1)^1,2^	1	–	–	1
Offspring of bull A (n = 84)	28 (33.3%)	5	6	17
Offspring of bull B (n = 105)	16 (15.2%)	5	3	8
Offspring of bull C (n = 66)	2 (3.0%)	1	1	0
Other direct and indirect	6	2	0	4
offspring of dam J (n = 8)	(75.0%)			
Total (n = 265)	54 (20.4%)	14	10	30

^1^Bull A had bilateral fistula but is not listed here because he was described elsewhere (Braun et al. [9]).

^2^Bull C was no longer alive at the time of this study.

### Examination

The eyes and periocular region were examined for the presence of unilateral or bilateral lacrimal fistula in all animals. The following were recorded for each fistula: location, size, shape, visibility, pigmentation, presence of hair, signs of inflammation and flow of tears. The location of the fistula was recorded in relation to the medial canthus of the eye using the clock face method with the medial canthus as the centre of the clock face. The location was then described as o’clock time. The height and length of each fistula was measured with a ruler, and the shape of the opening (circular, oval, slit-like) was recorded. The visibility of the fistula was scored as good when the opening could be seen from a distance of one metre without prior close examination of the animal. The visibility of the fistula was deemed poor when the opening was only recognised during closer examination or only after cleansing or stretching of the surrounding skin. Finally it was determined whether the fistula was inducing inflammation.

### Digital image documentation

Each fistula was photographed using a digital camera (Sony DSLR-A700, Sony Overseas SA, Schlieren) with a macro lens (Sony SAL100M28, Sony Overseas SA) and ring flash (Metz 15 MS-1, Metz-Werke GmbH & Co KG, Zirndorf) for complete documentation of all anomalies [10].

### Statistical analysis

The Stata software (StataCorp., 2011; Stata Statistical Software: Release 12; College Station, Texas, USA) was used to calculate means, standard deviations and frequency distributions. Continuous variables were tested for normality using the Shapiro-Wilk test. Differences of normal variables were analysed with a t-test. The Ladder test (Stata) was used to find the best transformation for non-normal data. Because meaningful transformations were not found for most variables, these were analysed using the Kruskal-Wallis rank sum test. A P-value ≤ 0.05 was considered significant.

## Results

### Affected cattle

**Figure 1:**
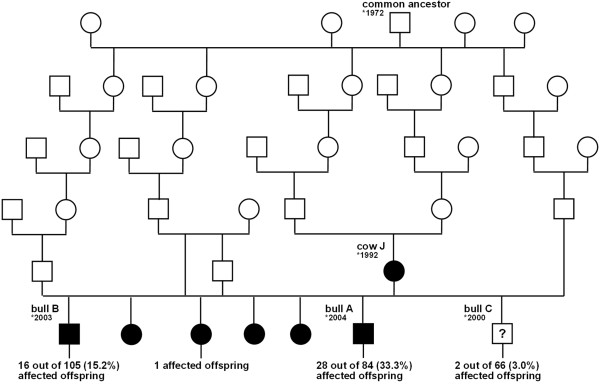
**Pedigree chart.** The pedigree chart showing the close relationship between the affected animals (shown with black symbols). Note that all cases can be traced back on the paternal and the maternal path to a common male ancestor born in 1972 indicating possible monogenic recessive inheritance.

Of 265 examined cattle, 54 (20.4%) had unilateral or bilateral nasolacrimal duct fistula (Table 1). The affected animals were 8 days to 19.3 years of age (median, 490 days). The fistula was unilateral in 24 animals; the left eye was affected in 14 and the right in 10. Bilateral fistula occurred in the remaining 30. Dam J had a fistula on the left, and bull B was affected bilaterally. The prevalence of affected offspring differed significantly among bulls A (28 of 84, 33.3%), B (16 of 105, 15.2%) and C (2 of 66, 3.0%) (Table 1, Figure 1). Six of the surviving eight (75.0%) direct or indirect offspring of dam J were affected.

### Location, size and shape of the fistula

All fistulae were medial to the medial canthus of the eye and the location relative to the latter varied little. Of the 44 fistulae on the left, 27 were at 8 o’clock, 14 at 9 o’clock and three at 7 o’clock, and of the 40 fistulae on the right, 21 were at 4 o’clock, 17 at 3 o’clock and two at 5 o’clock.

The size of the fistulae varied from 1 to 10 mm (median, 1 mm) in height and from 1 to 12 mm (median, 2 mm) in length (Table 2). Offspring of bull A had significantly higher (1 to 10 mm; median, 2 mm) and longer (1 to 12 mm; median, 2 mm) fistulae than those of bull B (height, 1 to 3 mm; median; length, 1 to 3 mm, median, 1 mm) (P < 0.05, Kruskal-Wallis rank sum test).

**Table 2:** **Height and length of 85 nasolacrimal duct fistulae in 52 cattle**^**1 **^**(median, range)**

	**Height (mm)**		**Length (mm)**	
**Animals**	**Left**	**Right**	**Left**	**Right**
Pedigree cow J (n = 1)	2 n = 1	–	5 n = 1	–
Bull B (n = 1)^2,3^	1 n = 1	1 n = 1	1 n = 1	1 n = 1
Offspring of bull A (n = 26)	2 (1 – 5) n = 20	2 (1 – 10) n = 21	2 (1 – 10) n = 20	2 (1 – 12) n = 21
Offspring of bull B (n = 16)	1 (1 – 3) n = 13	1 (1 – 2) n = 11	1 (1 – 3) n = 13	1 (1 – 3) n = 11
Offspring of bull C (n = 2)	1 n = 1	2 n = 1	1 n = 1	2 n = 1
Other direct and indirect offspring of dam J (n = 6)	2 (1 – 8) n = 6	2 (1 – 7) n = 4	2 (1 – 4) n = 6	2 (1 – 5) n = 4
Total (n = 52)	1 (1 – 8) n = 42	1 (1 – 10) n = 38	2 (1 – 10) n = 42	2 (1 – 12) n = 38

^1^Four fistulae of two daughters of bull A could not be measured for technical reasons.

^2^Bull A had bilateral fistula but is not listed here because he was described elsewhere (Braun et al. [9]).

^3^Bull C was no longer alive at the time of this study.

**Figure 2:**
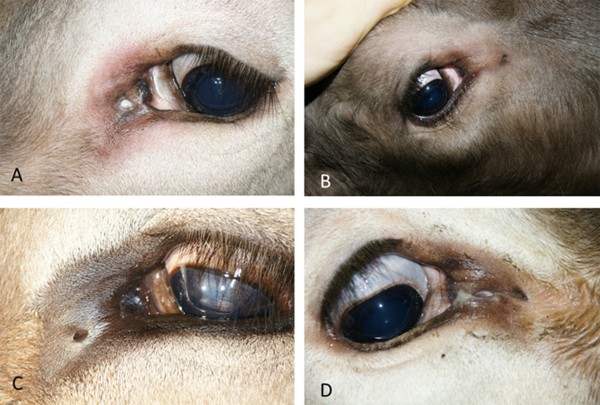
**Lacrimal duct fistulae with different-shaped openings. A)** Circular opening at 9 o’clock in the left ocular region of an eight-month-old daughter of bull A; **B)** Circular opening at 4 o’clock, 3.5 cm from the medial canthus in the right eye of a four-month-old daughter of bull B; **C)** Oval opening 1 cm medial to the medial canthus of the left eye in a two-year-old daughter of bull A; **D)** Slit-like opening 1.1 cm medial to the medial canthus of the right eye in a two-year-old daughter of bull A.

**Figure 3:**
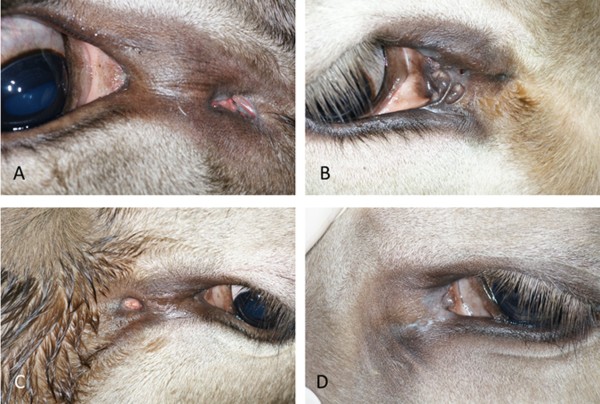
**Atypical openings and pigmentation of lacrimal duct fistulae. A)** Large atypical opening 2 cm medial to the medial canthus of the right eye in a two-year-old daughter of bull A; **B**: Severely abnormal medial canthus and several fistulous openings of the right eye a in a one-year-old daughter of bull A; **C)** Nasolacrimal duct fistula with a pigmented oval opening in the left eye of a two-month-old daughter of bull A; **D)** Nasolacrimal duct fistula with a non-pigmented oval opening in the left eye of a seven-month-old daughter of bull A.

Fifty-eight fistulae were circular (Figure 2A, B), 23 were oval (Figure 2C) and three were slit-like (Figure 2D). One other had a large atypical shape (Figure 3A), and one animal had a highly abnormal medial canthus with several fistulous openings (Figure 3B).

### Visibility, pigmentation and covering of hair

Visibility of the fistula was good in 50 (59.5%) and poor in 34 (40.5%). In some bilaterally affected animals, visibility was good in one and poor in the other fistula. The offspring of the three bulls did not differ with respect to visibility of the fistulae.

**Figure 4:**
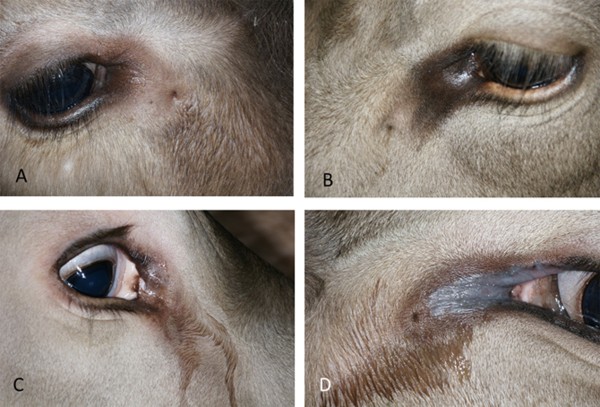
**Presence of hair and inflammatory changes at the openings of lacrimal duct fistulae. A)** Hairless nasolacrimal duct fistula at the transition from hairless to haired skin at 4 o’clock, 1.8 cm from the medial canthus of the right eye in a four-month-old granddaughter of dam J; **B)** Nasolacrimal duct fistula, covered with hair, at 8 o’clock, 4.5 cm from the medial canthus of the left eye in a four-year-old daughter of bull C; **C)** Nasolacrimal duct fistula with a circular opening with lacrimation in the right eye of a six-month-old daughter of bull A; **D)** Nasolacrimal duct fistula with a circular opening accompanied by inflammation of the skin and lacrimation in the left eye of a one-year-old daughter of bull A.

Seventy (83.3%) fistulae were pigmented (Figure 3C) and the remaining fistulae were nonpigmented (Figure 3D). 52 (61.9%) were hairless (Figure 4A) and the remaining fistulae were haired (Figure 4B). There was no difference among the offspring of the three bulls and between fistulae on the left and right side with respect to pigmentation and presence of hair. Hairless openings were mainly near the hairless region of the medial canthus (Figure 4A), and those covered with hair were all small, had poor visibility and were generally further away from the eye and thus surrounded by normally haired skin (Figure 4B).

### Flow of tears and inflammatory changes

Flow of tears was observed in 21 (25.0%) of the fistulae (Figure 4C) and was substantial in 10 and mild in 11. The secretion was serous in 19 cases, mucoid in one and purulent in one other. In one animal, the bilateral fistulae were associated with inflammatory changes in the form of hairless bluish-grey skin at the medial canthus (Figure 4D). The fistula affected the well-being in 12 animals because of severe lacrimation (n = 10), bilateral inflammation of the skin surrounding the fistulae (n = 1) and purulent material released from the fistula (n = 1).

## Discussion

Our study has shown that 20.4% of the female offspring of three bulls affected with fistula of the nasolacrimal duct had the same anomaly, albeit with frequencies that differed from 3.0% to 33.3%, depending on the bull. Congenital lacrimal duct fistula is a rare anomaly in people and can originate from the lacrimal canaliculi, lacrimal sac or nasolacrimal duct [3]. They usually manifest as small openings or dimples below and/or medial to the medial canthus of the eye. Congenital bilateral nasolacrimal duct fistula with a clinical manifestation similar to those described in this report was recently described in a four-year-old boy [3]. Our observations also agreed with those reported almost 40 years ago in 13 Brown Swiss calves in the USA, which had bilateral abnormal openings in the proximal third of the nasolacrimal duct at varying distances from the medial canthus of the eye [7]. All fistulae were patent and communicated with the respective nasolacrimal duct. Twelve were bilateral and one was unilateral. Contrast radiography of the head of one of the calves revealed three accessory ducts that communicated with the nasolacrimal duct and were up to 1.75 cm long. Slightly more than half of our cases were readily identified, which included primarily those fistulae that measured several millimetres in diameter or were accompanied by conspicuous hairlessness, regardless of their size. Possible reasons for the poor visibility of the remaining fistulae included, in addition to small size, presence of hair, skin folds and dirt or crusts of secretion that covered the opening. The relatively large proportion (40.5%) of fistulae with poor visibility demonstrates the necessity for a close and thorough examination, without which several of our cases would have gone undetected. The flow of tears from the fistulous openings seen in about a quarter of our cases may have been caused by obliteration of the nasolacrimal duct distally or may have occurred despite a normal patent nasolacrimal duct. Some of the fluid from the fistulae may have been attributable to inflammation. In 12 of 54 affected cattle (22.2%), lacrimal fistula induced lacrimation, inflammation and purulent discharge.

The pedigree of affected cattle was not analysed in a previous report on nasolacrimal duct fistula but the anomaly was believed to be hereditary [7]. Likewise, lacrimal fistula in people may be inherited as an autosomal dominant [11] or autosomal recessive trait [12]. Nasolacrimal duct fistula is often accompanied by other related anomalies and is therefore assumed to be a genetically heterogeneous condition [3]. We strongly suspect that the anomalies described in the present report have genetic etiology. Because the anomaly had a significantly different prevalence among the offspring of three affected bulls, it is suspected that the trait has variable expressivity and/or the mutation is modified by other mutations, rather than being a simple recessive trait with complete penetrance. How the presumed defective allele was introduced into the Brown Swiss cattle population in Switzerland via dam J or her ancestors remains unclear; however, during our study nasolacrimal duct fistula was incidentally identified in two non-related cows of the same breed, suggesting that this trait is not limited to the family of dam J. A spontaneous dominant germline-dependent mutation in dam J is therefore not likely responsible for the cases described in this study. Previous observations also suggested that lacrimal fistula in Brown Swiss calves is a recessive trait with a rare occurrence [7]. Interestingly, bilateral nasolacrimal duct fistula has also been reported in a Holstein calf [8], and during our study we identified an incidental Holstein cow with a unilateral nasolacrimal duct fistula. It is indisputable that extensive use of valuable breeding bulls in artificial insemination is responsible for increased milk production, but it also results in a decrease in genetic variation [13] and contributes to the distribution of gene defects in the population. As a result, the risk of autosomal recessive diseases increases.

## Conclusions

The findings suggest a hereditary cause of nasolacrimal duct fistula in Brown Swiss cattle. For zootechnical as well as health reasons, the spread of nasolacrimal duct fistula in the cattle population must be avoided. Molecular studies are under way to clarify the genetic background of this trait, which should aid in the identification of phenotypically normal carriers so that the use of carrier animals in breeding programs can be avoided.

## Competing interests

The authors declare that they have no competing interests.

## Authors’ contributions

UB initiated, planned and supervised the study and wrote the manuscript, SJ completed the study, CD was involved in the planning of the study, selection of cattle and supervision of SJ. All authors have read and approved the manuscript.

## Fundings

This study was financed by the University of Zurich, Switzerland.
